# Separation of photoreceptor cell compartments in mouse retina for protein analysis

**DOI:** 10.1186/s13024-017-0171-2

**Published:** 2017-04-11

**Authors:** Kasey Rose, Steven T. Walston, Jeannie Chen

**Affiliations:** 1 Zilkha Neurogenetic Institute, Keck School of Medicine, University of Southern California, Los Angeles, California USA; 2 Department of Biomedical Engineering, Viterbi School of Engineering, University of Southern California, Los Angeles, California USA; 3 Department of Cell & Neurobiology, Keck School of Medicine, University of Southern California, Los Angeles, California USA

**Keywords:** Retina, Protein translocation, Protein trafficking, Phototransduction, Transducin, Arrestin, RGS9

## Abstract

**Background:**

Light exposure triggers movement of certain signaling proteins within the cellular compartments of the highly polarized rod photoreceptor cell. This redistribution of proteins between the inner and outer segment compartments affects the performance and physiology of the rod cell. In addition, newly synthesized phototransduction proteins traverse from the site of their synthesis in the inner segment, through the thin connecting cilium, to reach their destination in the outer segment. Processes that impede normal trafficking of these abundant proteins lead to cell death. The study of movement and unique localization of biomolecules within the different compartments of the rod cell would be greatly facilitated by techniques that reliably separate these compartments. Ideally, these methods can be applied to the mouse retina due to the widespread usage of transgenic mouse models in the investigation of basic visual processes and disease mechanisms that affect vision. Although the retina is organized in distinct layers, the small and highly curved mouse retina makes physical separation of retinal layers a challenge. We introduce two peeling methods that efficiently and reliably isolate the rod outer segment and other cell compartments for Western blots to examine protein movement across these compartments.

**Methods:**

The first separation method employs Whatman^®^ filter paper to successively remove the rod outer segments from isolated, live mouse retinas. The second method utilizes Scotch^TM^ tape to peel the rod outer segment layer and the rod inner segment layer from lyophilized mouse retinas. Both procedures can be completed within one hour.

**Results:**

We utilize these two protocols on dark-adapted and light-exposed retinas of C57BL/6 mice and subject the isolated tissue layers to Western blots to demonstrate their effectiveness in detecting light-induced translocation of transducin (GNAT1) and rod arrestin (ARR1). Furthermore, we provide evidence that RGS9 does not undergo light-induced translocation.

**Conclusions:**

These results demonstrate the effectiveness of the two different peeling protocols for the separation of the layered compartments of the mouse retina and their utility for investigations of protein compositions within these compartments.

**Electronic supplementary material:**

The online version of this article (doi:10.1186/s13024-017-0171-2) contains supplementary material, which is available to authorized users.

## Background

**Fig. 1:**
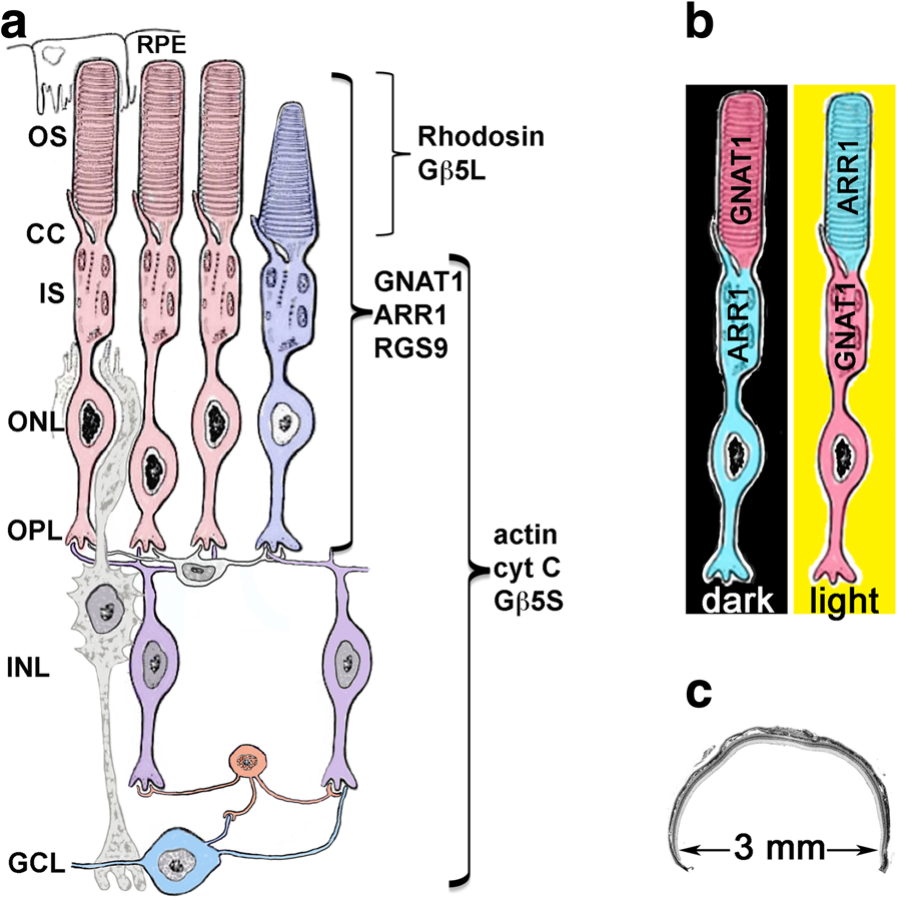
Diagram of retinal cell layers in the mouse retina. **a** Retinal layers and associated cell types: rod (*pink*), cone (*purple*), bipolar (*lilac*), Müller (*gray*), ganglion (*blue*) cells. RPE: retinal pigmented epithelium, OS: outer segment, CC: connecting cilium, IS: inner segment, ONL: outer nuclear layer, OPL: outer plexiform layer, INL: inner nuclear layer, GCL: ganglion cell layer. Rhodopsin and Gβ5L are localized to the OS. GNAT1 (rod transducin α-subunit), ARR1 (rod arrestin) and RGS9 are also localized in rod cells. Actin, cytochrome C (cyt C) and Gβ5S are expressed in all retinal layers except the OS. **b** GNAT1 and ARR1 are localized to different rod cell compartments under different lighting conditions. **c** The dimension of a central cross section from the posterior pole of the mouse eye containing the neural retina

Rod photoreceptor cells are highly polarized and specialized sensory neurons that convert photon absorption into neural signals [1]. Each rod cell has a distinct morphology that is composed of an outer segment (OS), an inner segment (IS), a cell nucleus residing in the outer nuclear layer (ONL), and a synaptic terminal located at the outer plexiform layer (OPL). Each of these compartments is aligned in the layered structure of the retina (Fig. 1a), and each contains unique molecular signatures and protein complexes [2–4]. The rod outer segment (ROS) consists of tightly stacked membranous discs wherein the light-sensitive G-protein coupled receptor, rhodopsin, is embedded in high density [5]. Also in the OS are other membrane proteins, membrane-associated and soluble proteins that are important for phototransduction and for the structural integrity of the OS [2].

Phototransduction begins with photon absorption by 11-cis retinal, the visual chromophore covalently attached to rhodopsin [1]. Light-activated rhodopsin catalyzes GDP-GTP exchange in multiple transducin molecules. Rhodopsin deactivation occurs in two steps: First, rhodopsin kinase (GRK1) places multiple phosphates on the receptor’s carboxyl-terminus [6–10]. Second, arrestin (ARR1) binds to activated, phosphorylated receptor, which fully blocks transducin activation [11, 12]. Transducin-GTP binds to the inhibitory subunit of phosphodiesterase 6 (PDE6), releasing its catalytic activity for cGMP hydrolysis [13, 14]. Upon reduction of cGMP concentration, the cGMP-gated channels close, reducing the influx of cations [15–17]. The change in current hyperpolarizes the cell and reduces glutamate release at the synaptic terminus.

As early as the 1980’s, light-triggered movement of transducin away from, and ARR1 movement towards the ROS have been observed using immunocytochemistry [18–21] (Fig. 1b). This movement of the two key phototransduction proteins that have opposing actions-in opposite directions-was expected to have significant physiologic consequences. However, immunocytochemistry is susceptible to epitope masking and thus observation of this curious phenomenon was received with some skepticism [22]. On the other hand, although epitope masking may not be an issue for biochemically isolated ROS, caveats for this methodology for the investigation of protein translocation include 1) the procedure is lengthy, 2) protein may leak out of ROS during mechanical breakage of ROS from the thin connecting cilium (Fig. 1a, CC) and 3) a sizable amount of retinal isolate is required. It was not until the early 2000’s when physical separation of the layered photoreceptor cell compartments using tangential sectioning of rat retinal flat mounts, followed by protein immunoblots of sequential sections, that the field of light-driven protein translocation experienced its renaissance [23–28]. The study by Sokolov et al. [23] demonstrated that up to 90% of transducin leaves the ROS following light exposure. The resulting reduction in sensitivity was proposed to be a mechanism for light adaptation, and is also protective against cell death induced by excessive signaling [29]. Additional reports on ARR1 translocation indicated that such movement was protective against light damage [30–32]. Despite the rigor of tangential sectioning in the demonstration of unique protein signatures in distinct rod compartments, the intrinsic curvature of the rodent retina makes reliable orientation of the flat mounted tissue for collection of orthogonal sections difficult.

Although many mouse models have been created for the investigation of basic visual processes and human retinal diseases, the mouse eye is small and highly curved (Fig. 1c), with the retinal area 3-fold smaller than that of the rat retina. These factors make serial tangential sectioning heavily operator dependent, which results in a steep learning curve for the technique. In this study, we introduce two peeling methods to address this challenge (see Additional file 1). The first involves sequential removal of live retinal tissue by filter paper, and the second utilizes adhesive tape to separate retinal layers in a lyophilized retina. With small sample size, both methods can be completed within an hour. The separated layers, even from a single retina, provide sufficient material for certain biochemical analyses. We provide validation for these methods by Western blots of isolated retinal layers using protein markers for different rod compartments (Fig. 1a).


Additional file 1: (MP4 358510 kb)


## Methods

### Animals

All experiments were conducted using non-breeder male and female C57/B6 mice (2–3 months old). Each sex contributed to roughly half of the total animal number in each experiment. Animals were housed in a 12/12 h dark/light cycle and had unrestricted access to food and water. The use of mice in these experiments was in accordance with the Guide for the Care and Use of Laboratory Animals and experimental protocols were approved by the University of Southern California Institutional Animal Care and Use Committee (IACUC).

### Light exposure

Eyes were dilated with 0.5% Tropicamide Ophthalmic Solution, USP (AKORN) and 2.5% Phenylephrine Hydrochloride Ophthalmic Solution, USP (AKORN). The mice were dark-adapted overnight. They were kept in darkness or exposed to a diffuse cool white fluorescent light at luminescence level of 5000 lux for 30 min to 1 h prior to being euthanized. The dark-adapted samples for both methods were prepared in a darkroom under infrared light and all procedures involving dark-adapted tissue were performed using a dissecting microscope fitted with infrared converters (B.E. Meyers & Co, Inc.). The light exposed samples were processed under a dissecting scope in room light.

### Retina dissection

Mice were euthanized by isoflurane inhalation followed by cervical dislocation. The eyes were enucleated and the retinas were isolated in a 35 × 10 mm petri dish filled with the appropriate buffer/solution described below. The cornea, lens, and vitreous humour were removed from each eye and the retinal pigmented epithelium (RPE) and sclera were carefully peeled away from each retina. The isolated retinas were hemisected with a feather scalpel in a 60 × 15 mm dish and the edges were trimmed to engender two rectangles. Minimizing the curvature of each halved retina assisted in flattening the retina and ensured an accurate peel of retinal layers. Proper trimming of the folding edges of the retina is essential: if the retina has folding edges when placed on the filter paper, it will result in a decreased yield of isolated ROS and more importantly will be contaminated with other retinal layers.

### Immunocytochemistry

Before enucleation, the superior pole of the cornea was marked by cauterization and the cornea, lens, and vitreous were subsequently removed. The remaining eye cups were placed in 4% paraformaldehyde in PBS for 15 min and rinsed 3 times for 10 min in PBS. The eye cups were cryoprotected in 30% sucrose in PBS for 2 h, placed in Tissue-Teck® O.C.T. compound (Sakura Finetek, USA) and quickly frozen in liquid N_2_. The frozen blocks were sectioned at 10 μm in a cryostat (CM 3050 S, Leica Microsystems) and stored in −80 °C. Prior to antibody incubation, the sections were equilibrated to room temperature (RT) for 15 min. For GNAT1 staining using the TF-15 mouse monoclonal antibody, epitope retrieval was performed: sections were treated for 2 min RT with 0.02 mg/ml proteinase K in blocking buffer (2% bovine serum albumin, 2% goat serum, 0.3% Triton X-100 in 1X PBS) and heated to 65 °C for 10 s followed by five rinses with PBS. Blocking buffer was then applied to all sections for 1 h. Sections were either incubated with the rabbit antibody against ARR1 (C10C10 [33, 34] diluted 1:100 in blocking buffer) or TF-15 (CytoSignal, diluted 1:200 in blocking buffer). The sections were rinsed and incubated with a fluorescein-labeled secondary antibody (Vector Laboratories). All sections were then double-stained with the biotinylated antibody against rhodopsin (1D4 [35] diluted 1:300 in blocking buffer). The sections were rinsed and incubated with rhodamine Avidin D (1:100, Vector Laboratories). Images were obtained using a Zeiss AxioPlan2 microscope. Light and dark conditions were imaged using identical exposure times.

### ROS collection by sequential peeling with filter paper

The filter paper peeling method was adapted from a technique to expose fluorescently tagged bipolar cells in a retinal flat mount for patch clamp recordings [36]. Peeling away the photoreceptor cell’s multiple layers with filter paper gradually exposes the bipolar cell dendrites and cell bodies for easier access for electrical stimulation and patch clamp readings. We found that the peeled byproducts, the photoreceptor layers that are stuck on the filter paper, were amenable for subsequent Western blot analyses.

Ames’ medium was used for manipulation of live retinal tissue (Sigma-Aldrich A1420). Two different buffers were prepared: Ames’-HEPES (to 1 L add 2.38 g HEPES, 0.877 g NaCl, pH 7.4) and Ames’-bicarbonate (to 1 L add 1.9 g NaHCO_3_, pH 7.4). Both were prepared in advance, sterile filtered, and stored at 4 °C. All procedures were performed at RT. Prior to retinal dissection, 50–100 mL of Ames’-HEPES was bubbled with 100% O_2_ in a Gibco^TM^ 100 mL media bottle (Thermo Fisher, USA) and 50–100 mL of Ames’-bicarbonate was bubbled with 95% O_2_ and 5% CO_2_ in a light-tight container for 15–20 min before use.

Retinas were prepared as described in the ‘Retina dissection’ section and stored in a light tight container with Ames’-bicarbonate bubbled with 95% O_2_ and 5% CO_2_ to maintain physiological pH. This incubation condition is identical to that used by retinal physiologists for ex vivo electroretinogram recordings or suction electrode recordings [37], and can maintain tissue viability and functionality for several hours. A halved piece of rectangular trimmed retina was used for each peeling procedure. The tissue was transferred via a 1.7 mL plastic transfer pipet (tip cut) into a 35 × 10 mm petri dish containing oxygenated Ames’-HEPES. The media was refreshed every 10 min during the peeling process to maintain the oxygenated state. The retina in solution was oriented with the photoreceptor side facing down using tweezers and the transfer pipet. A 5 mm × 2.5 mm rectangular piece of filter paper cut from VWR grade 413 filter paper (diameter 5.5 cm, pore size 5 μm, VWR, USA) was placed into the petri dish next to the retina. The retina was carefully moved with tweezers (lightly holding the edges) onto the filter paper with the photoreceptor side down. Once the retina was centered on the filter paper, both were carefully lifted out of the Ames’-HEPES. The bottom side of the filter paper (the side without the retina) was blotted on paper towel to soak up the liquid on the filter paper (2–3 dabs). This created a secure adhesion between the photoreceptor cells and the fibers of the filter paper, and this attachment was important for removing the ROS layer. A drop of Ames’-HEPES from the petri dish was placed on the retina, and the filter paper blotted again on the paper towel. This was repeated a total of three times. The filter paper with the retina was then placed back into the petri dish and submerged, and the tissue removed from the filter paper with tweezers. Care was taken to touch only the extreme perimeter of the retina to preserve the retina’s structural integrity. To facilitate the peeling process, the edges of the retina were gently peeled away from the filter paper from each side, loosening the adhesion of the retina to the filter paper. Once the retina was removed from the filter paper, the bottom surface of the filter paper was blotted on a paper towel and placed into a tube labeled +ROS and was kept on ice. The peeling process described above was repeated approximately 7–8 times. After 5 peels, the retina became thinner, more transparent, and was prone to tear. After peeling, the +ROS tube containing the collected filter papers and the remaining peeled halved retina was placed in the -ROS tube, frozen on dry ice and stored at −80 °C.

### Separation of photoreceptor compartments by peeling lyophilized retina

This method was adapted from that described by M.E. Guido, et al. [38], who designed a Scotch^TM^ tape peeling method that utilized lyophilized chick retinas to selectively separate the retina into different layers (photoreceptor cells, inner nuclear layer, and ganglion cells). Since retinas from different animal models have different rod and cone distributions and may separate asymmetrically with tape after lyophilization, we adapted this method and explored its utility for the separation of rod compartments of mouse retinas.

#### Freeze-drying of isolated retinas

Retinas were prepared as described in ‘Retinal dissection’ section. Cold Ringer’s (130 mM NaCl, 3.6 mM KCl, 2.4 mM MgCl_2_, 1.2 mM CaCl_2_, 10 mM HEPES, 0.02 mM EDTA, pH 7.4) was used during the dissection. Other physiologic buffers, such as Ames’-HEPES, could also be used. Because multiple samples were often handled at the same time, the 5 × 2.5 mm filter paper pieces (Whatman® Grade 1 Qualitative filter paper (diameter 9 cm, pore size 11 μm, GE Healthcare, USA)) were labeled ahead of time before they were placed into a petri dish filled with cold Ringer’s buffer. For each sample, a halved, rectangular piece of retina was positioned on the filter paper using a 1.7 mL transfer pipet (cut tip) with the ganglion cell side down and the photoreceptor outer segment side up. Once the tissue was centered on the filter paper, both were lifted out of the Ringer’s buffer and the bottom of the filter paper (the side without the retina on it) was blotted on a paper towel (2–3 dabs) to facilitate attachment of the ganglion cell layer onto the filter paper. A drop of cold Ringer’s was placed on the retina, and the filter paper bottom was again blotted on the paper towel. This was repeated a total of three times. Ringer’s buffer was swapped out for new cold Ringer’s buffer after each sample preparation. The filter paper with attached retina was placed in a petri dish filled with cold Ringer’s until all samples were processed in the same fashion. Finally, each was again lifted out of the solution, the bottom of the filter paper blotted dry, and a drop of cold PBS placed on the filter paper next to the retina, the bottom of the filter again blotted, and placed into a clean and dry 35 × 10 mm petri dish. The purpose of this step was to rinse off the more complex Ringer’s with PBS to reduce the amount of dried salt on the lyophilized tissue. After all tissue samples had been processed with this final rinse step and collected into the clean petri dish, the dish was wrapped light tight with two layers of 2.5 × 2.5 inch square pieces of aluminum foil, with small holes, so that the dark-adapted retina samples are not exposed to light, and quickly frozen in liquid N_2_. The small holes allowed liquid N_2_ access into the interior, filling the petri dish, and care was taken to ensure that the holes were offset so that the petri dish was wrapped light-tight. The petri dish was then placed in a 600 mL Labconco flask using a VirTis Benchtop 2 K Lyophilizer (SP Scientific, USA) for 30 min to lyophilize the tissue.

#### Peeling of retinal layers by Scotch^TM^ tape

Freeze-dried retinal tissues were stored at −80 °C in a Drierite^TM^ (W.A. Hammond Drierite Co, USA) filled container or were isolated as +ROS, +RIS, and -ROS/RIS-depleted tissue (-OIS), frozen again as above or processed for Western blots the same day. All peeling procedures were performed under room light. Strips smaller than 2.5 mm in width were cut and placed on the edge of the tape dispenser until trimmed into rectangular pieces. A lyophilized retina fixed to Whatman® filter paper was placed in a clean 10 cm petri dish. A small rectangular piece of Scotch^TM^ tape (slightly larger than the surface of the tissue) was cut and carefully laid on top of the lyophilized retina. Placing the tape on top of the lyophilized retina was almost sufficient to attach the orange-tinted ROS layer to the tape. To ensure complete contact of the ROS with the tape, slight pressure was applied with tweezers to the top of the tape to ensure contact with the top surface. After carefully peeling away the tape, the orange-tinted ROS layer was adhered to the tape and separated from the rest of the retina. This fraction was labeled +ROS and placed into a clean microfuge tube. Often, a thin, white film was visible at the fractured surface of the orange layer on the first tape peel. This surface was removed by more tape peels until it was completely removed and the orange color of the ROS layer was brought to the surface. This white layer initially attached to ROS was placed into a separate tube and labeled +RIS fraction. Tape was used to remove the leftover retinal tissue from the filter paper and was placed into a tube labeled -OIS. The amount of pressure applied to the tape for the initial peel of the orange-tinted ROS layer affected how the lyophilized sample fractioned; too much pressure caused the whole lyophilized retina to peel off the filter paper onto the tape and too little pressure did not separate the top orange layer from the retina. A couple of passes over the tape using minimal pressure with tweezers was helpful in feeling out the minimum and maximum amount of pressure to add to the top of the tape.

### Sample preparation for Western blot: peeling with filter paper

The +ROS tubes containing the filter papers were subjected to a quick spin in a microcentrifuge for 2 s and the excess liquid removed. Each tube was processed singly (one halved retina) or two tubes (two halved retinas) were combined for more concentrated material. The +ROS isolate in a single tube was homogenized in 45–60 μL of cold RIPA buffer buffer (50 mM Tris–HCl pH 7.4, 150 mM NaCl, 1% Triton X-100, 1% sodium deoxycholate, 0.1% SDS, 1 mM EDTA, 0.1 M PMSF, complete mini protease inhibitor (Roche Applied Sciences)), and the -ROS isolate in a single tube was homogenized in 80–100 μL RIPA buffer. When two tubes were combined, 80–110 μL of cold RIPA buffer was used to homogenize +ROS and 100–150 μL of cold buffer was used for -ROS. All tubes were homogenized for 1 min with an autoclaved pestle. Great care was taken to make sure that the filter paper pieces in the +ROS tubes were kept on the side of the tubes and stayed in contact with the pestle instead of being stuck at the bottom. After homogenization, sterile tweezers were used to move the filter paper pieces to the side of the +ROS tube, followed by 2–4 s spin in the microcentrifuge. This spin extracted the liquid from the paper, and the liquid was transferred into a clean tube and processed for Western blot as described below. This was the most time-consuming step, and if not performed properly, much of the sample may end up being absorbed by the pieces of filter paper. To maximize recovery of sample, the size of the filter paper pieces used for the peels should be trimmed to match the area of the retinal tissue.

### Sample preparation: peeling with Scotch^TM^ tape

Similar to the filter peeling method, two tubes, each containing a peeled layer from one-half retina, were combined to increase protein concentration. +ROS and +RIS samples were homogenized in 100–115 μL, and -OIS samples in 125 μL of cold RIPA buffer. All tubes were homogenized for 1 min. Great care was taken to make sure that the tape in the +ROS, +RIS, and -OIS tubes stayed in contact with the pestle and that the tape was kept on the side of the tubes instead of being stuck at the bottom. After homogenization, sterile tweezers were used to move pieces of tape to the side of the tubes. The tubes were spun in the mini centrifuge for 2–4 s, after which the dried pieces of tape were carefully removed.

### Protein quantification, gel electrophoresis and protein immunoblots

A BCA protein assay kit (Thermo Scientific, USA) was used to determine the total amount of protein in each sample. Two microliter of DNaseI (10 units/μL, Roche, Switzerland) were added to the samples and were left at room temperature for 30 min. Appropriate volume of 4X SDS sample buffer (40% glycerol, 240 mM Tris Base pH 6.8, 8% SDS, 5% βME, 0.04% Bromophenol blue) was added to the homogenate. The average protein yields for two halved retinas combined for both filter paper peel and lyophilized retina peels are as follows:

**Table:** 

**Filter peeling of live retina**				
	+ROS		-ROS	Whole retina
Average protein concentration (μg/mL)	700		1500	1500
RIPA volume (μL)	90		90	200
**Tape peeling of lyophilized retina**				
	+ROS	+RIS	-OIS	Whole retina
Average protein concentration (μg/mL)	520	440	1200	1600
RIPA volume (μL)	100	100	125	150

Approximately 10 μg of protein lysate was loaded per lane and separated on a pre-cast polyacrylamide Bis-Tris gel with a 4–12% gradient (Life Technologies, USA) and transferred onto nitrocellulose membrane. The membranes were blocked in 10% milk/TBS-T buffer for 1 h at RT, and incubated overnight with the following antibodies: rabbit anti-ARR1 antibody (1:1000, ref), mouse anti-GNAT1 monoclonal antibody (TF-15, 1:1000, CytoSignal), rabbit anti-β actin antibody (1:5000, GeneTex Inc.), rabbit anti-RGS9 antibody [39] (1:1000), rabbit anti-Gβ5L/S (CT215) [40] (1:2000), and rabbit anti-cytochrome C polyclonal antibody (1:500, Santa Cruz, sc-7159). Membranes were incubated with fluorescently labeled secondary antibodies (1:10,000, LI-COR Biosciences) at RT for 1 h. The protein bands were detected by Odyssey® infrared imaging system (LI-COR Biosciences, USA) and the fluorescence intensity of individual bands was quantified using ImageJ. GNAT1, ARR1 and RGS9 signals were normalized against Gβ5L for +ROS, +RIS samples, and against actin for -ROS and -OIS samples. For each independent experiment, fluorescent signals for each protein in each compartment were also normalized against combined signals from all retinal compartments. Unpaired 2-tailed *t*-tests were used to determine differences between two groups.

## Results

### Detection of light-induced protein translocation in isolated, live mouse retina by sequential peeling of retinal layers with filter paper

**Fig. 2:**
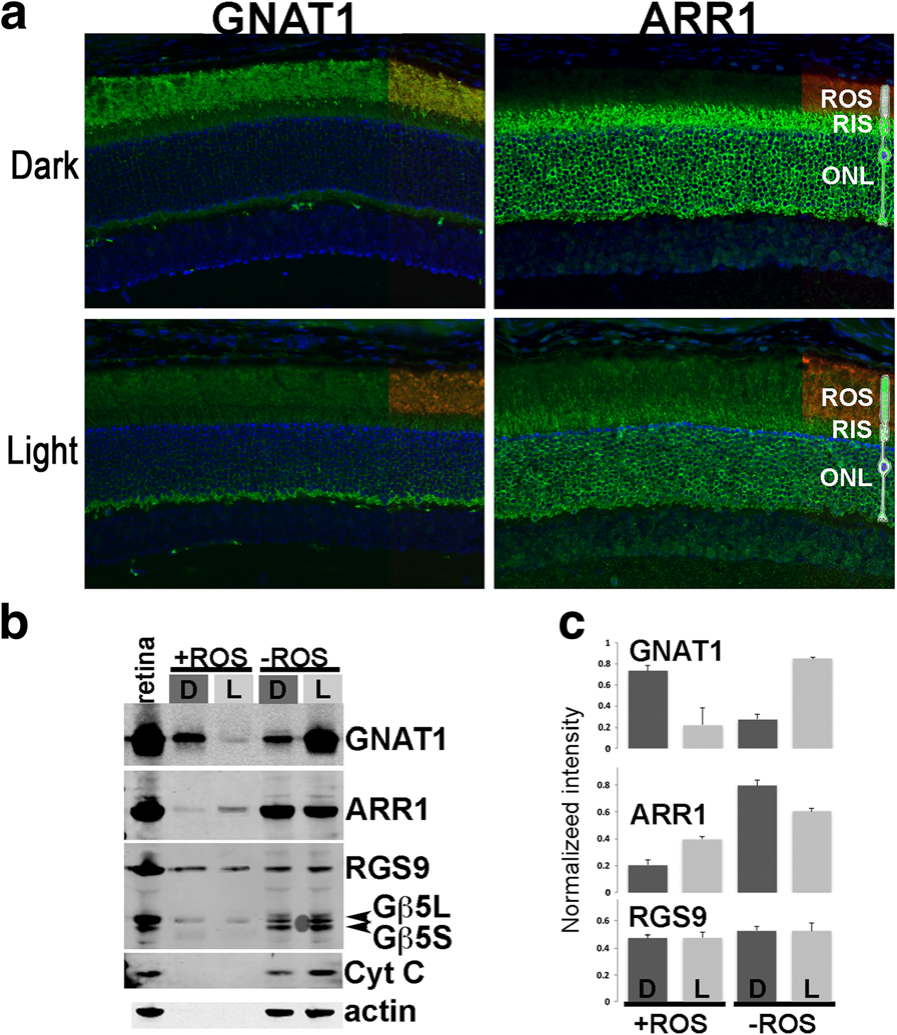
Light-induced movement of GNAT1 and ARR1 in rod photoreceptors. **a** Frozen retinal sections prepared from dark-adapted or light-exposed mice incubated with GNAT1 or ARR1 antibodies (*green*). The location of the rod outer segment is visualized with an antibody against rhodopsin (*red*), shown at the right of each panel. The diagram of the rod cell depicts the position of each rod compartment on the retinal section. Scale bar = 20 μm. **b** Representative immunoblots of ROS collected by filter paper peeling of retinas obtained from dark-adapted (D) or light exposed (L) mice. The -ROS fraction is the ROS-depleted tissue. **c** Quantified signals from light exposed (*n* = 6) and dark adapted (*n* = 5) +ROS and -ROS samples plotted as mean ± SD. There was a statistically significant difference between +ROS (D)/+ROS (L) (*p* < 0.0003) and -ROS (D)/-ROS (L) respectively for GNAT1 (*p* < 0.0001) and ARR1 (*p* < 0.0001) using unpaired *t*-test. There was no statistically significant difference between +ROS (D)/+ROS (L) and -ROS (D)/+ROS (L) for RGS9 (*p* = 0.8)

Light exposure was rigorously controlled by dark-adapting the animals overnight in a light-tight environment and all procedures performed under infrared light. To visualize the extent of translocation of these proteins under our light exposure protocol, we prepared retinal sections from dark-adapted and light exposed mice and incubated them with antibodies against the α-subunit of rod transducin (GNAT1) and rod arrestin (ARR1). As seen in Fig. 2a, GNAT1 immunoreactivity was most intense in the rod outer segment (ROS) in the dark-adapted retina, whereas ARR1 fluorescence is largely excluded from this space but strongest in the cytoplasm of more proximal compartments. This pattern was reversed in the light exposed retina: GNAT1 immunoreactivity shifted from ROS toward the proximal compartments, while ARR1’s immunoreactivity spread from the proximal compartments toward the ROS (Fig. 2a). Having validated the light exposure protocol’s effectiveness in triggering protein translocation, retinas were isolated from mice in darkness or after light exposure and were subjected to sequential peeling using filter paper. The filter papers containing the OS layer were pooled (+ROS), and signals from the indicated proteins were compared between +ROS samples and the remaining tissue (-ROS) using Western blots (Fig. 2b). Whole retinal homogenate served as the input control (Fig. 2b, retina). Gβ5L, a component of GAP for transducin [41], is localized to the ROS whereas the Gβ5S, the shorter splice isoform, is excluded from the ROS. Gβ5S is instead expressed in the proximal rod compartments as well as the remaining retinal layers [40] (Fig. 1a). Because Gβ5S is excluded from ROS, it also served as a quality control for ROS purity [37, 42]. As can be seen in Fig. 2b, no Gβ5S signal was observed in the +ROS samples, demonstrating an absence of contamination from other cellular layers in these samples. Other indicators of ROS purity included cytochrome C (Cyt C) and actin, both abundant proteins in the proximal rod compartments as well as other retinal cell layers, but absent in ROS (Fig. 2b). Together, these results confirm the purity of the ROS samples. In contrast, Gβ5S signal was clearly visible in the -ROS samples, and its relative level to Gβ5L increased when compared to that of total retinal homogenate (Fig. 2b). This result is consistent with a depletion of ROS from these samples. Consistent with the immunocytochemistry results (Fig. 2a), the dark-adapted, +ROS sample exhibited the strongest GNAT1 signal (Fig. 2b). This signal was noticeably reduced in the light exposed +ROS sample. This pattern was reversed in the -ROS sample, a result to be expected if ROS was successfully removed from the other retinal layers. Similarly, light-induced ARR1 translocation can be visualized in both +ROS and -ROS samples (Fig. 2b). Although the -ROS sample contained other retinal layers in addition to the photoreceptor cell layer, in this instance the data reflects the amount of GNAT1 and ARR1 in the proximal photoreceptor compartments as they are not expressed in other retinal cell layers. We also examined whether light exposure caused a shift in the levels of RGS9 within ROS and RIS. In contrast to a recent report [43], we did not detect any light-dependent changes for RGS9 in these compartments (Fig. 2b). Results from 6 experiments were quantified and plotted in Fig. 2c (mean ± SD). Statistically significant differences were found between light/dark conditions for GNAT1 (*p* < 0.0003) and ARR1 (*p* < 0.0001) in both +ROS and -ROS samples. No light/dark differences were found for RGS9 in either +ROS or -ROS samples (*p* = 0.7973). Together, these data demonstrate the effectiveness of the filter peeling method in separating ROS from other retinal layers. Furthermore, the results confirm light-induced movement of GNAT1 and ARR1, but not RGS9, in the rod photoreceptor ROS.

### Isolation of rod compartments by peeling of lyophilized mouse retinas using adhesive tape

**Fig. 3:**
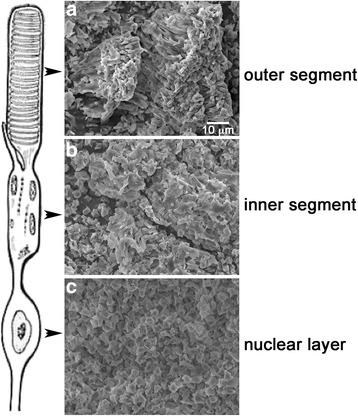
Scanning electron micrographs of lyophilized retina. **a** Surface of lyophilized retina, photoreceptor side up, before peeling by adhesive tape. **b** The inner segment layer beneath the ROS. **c** Photoreceptor cell nuclear layer shows the uniform appearance of rod cell nuclei

We also investigated a method for separating retinal layers in lyophilized mouse retinas by adapting procedures previously described for chick [38] and frog retinas [44]. To demonstrate that this method could be used to reproducibly isolate the ROS and RIS compartments in the small and highly curved mouse retina, we imaged the surfaces of the peeled lyophilized retinas using a scanning electron microscope (SEM). Retinas were placed on small filter paper squares, oriented with the ROS side up and quickly frozen in liquid N_2_ before placing in a Labconco flask and freeze-dried using a VirTis Benchtop 2 K lyophilizer (SP Scientific, USA). Before peeling, the surface of the intact lyophilized specimen showed clumps of the characteristic elongated cylindrical ROS throughout (Fig. 3a). After the first peel, the ROS and RIS structures appeared to be effectively removed, exposing the uniform structure of cell bodies of the ONL (Fig. 3c). Subsequent peels from the bottom of the first peel removed the RIS (Fig. 3b). The separation between the ROS and RIS is facilitated by their distinctive appearance: the ROS is thicker and orange in color whereas RIS is a thin white layer. The separation between ROS and RIS is also likely aided by their relatively weak attachment at the thin connecting cilium. These results from the SEM images indicate that retinal layers can be sequentially separated in lyophilized mouse retinas using adhesive tape.

**Fig. 4:**
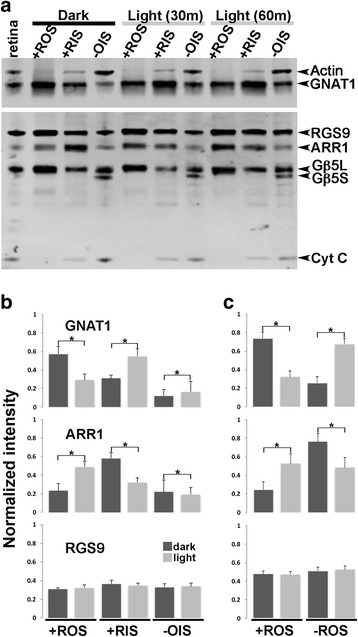
Western blots of retinal layers isolated by Scotch™ tape peels of lyophilized retinas. **a** Representative immunoblots from +ROS, +RIS and ROS/RIS-depleted tissue (-OIS). **b** Signals from the Western blots were quantified and plotted as mean ± SD for light (*n* = 9) and dark (*n* = 5) conditions. Dark (D) and light (L) samples showed statistically significant differences for +ROS, +RIS and -OIS for GNAT1 (*p* < 0.0001) and ARR1 (*p* < 0.0001) using unpaired *t*-test. Light/dark differences were not statistically significant in all samples for RGS9 (*p* = 0.2) **c** Signals from the lyophilized isolations of +RIS and -OIS were combined and plotted for comparison with the filter paper peeling method (*n* = 19 (L) and *n* = 10 (D)). Light and dark samples were found to be statistically different for +ROS and -ROS for GNAT1 (*p* < 0.0001) and ARR1 (*p* < 0.0001) respectively. There was no statistically significant difference between RGS9 light and dark samples (*p* = 0.7)

The separated layers from lyophilized retinas were subjected to Western blots using the same panel of molecular markers shown in Fig. 2. A representative Western blot of the isolated layers is shown in Fig. 4a, which also included a whole retinal homogenate input control. For this experiment, lyophilized retinas were prepared from mice kept in darkness or exposed to 5000 lux light for 30 min or 60 min. The +ROS samples showed signals for Gβ5L but not Gβ5S, and signals for both actin and cytochrome C were also absent (Fig. 4a). These results were similar to the +ROS samples shown in Fig. 2b and confirm the absence of contaminant from other retinal compartments in the lyophilized +ROS samples. The next layer, +RIS, contains mitochondria and, as expected, cytochrome C signal was detected in all +RIS samples (Fig. 4a). Interestingly, the +RIS samples did not contain Gβ5S, but did contain Gβ5L, albeit at a lower level when compared to +ROS samples. The remaining retinal layers (-OIS) had protein signatures similar to filter paper peeled -ROS samples shown in Fig. 2b. In terms of light-induced protein translocation, a clear shift of GNAT1 from +ROS to +RIS was observed. A shift to the remaining retinal layers was also detected, but the extent was not as dramatic as that from +ROS to +RIS (Fig. 4a). Similarly, a light-induced shift was clearly visible for ARR1: it was most abundant in the dark-adapted +RIS sample, and its signal shifted to +ROS in the light exposed samples. No differences were observed between 30 min and 60 min light exposed conditions, indicating a steady-state had been reached by 30 min under our light exposure protocol. Fig. 4b shows the quantified and normalized signals from the indicated proteins in the +ROS, +RIS and -OIS layers. Data from +RIS and -OIS were also combined and plotted (Fig. 4c) for comparison with Fig. 2c, which summarizes the filter paper peeling method. The graphs show the same profile of light-induced changes for GNAT1 and ARR1, but not RGS9. We conclude that both sequential filter paper peeling of live retina and adhesive tape peeling of lyophilized retina offer a rapid, reproducible alternative means for separating layers of the mouse retina for subsequent biochemical assays.

## Discussion

Rod photoreceptor cell death is a primary cause of blindness [45], and genetic defects that affect protein trafficking within the highly polarized rod cell are responsible for a large proportion of inherited retinal degeneration [46–50]. Defects in light-induced protein trafficking also affect the health and performance of rod photoreceptors [27]. The ability to separate rod compartments for biochemical/molecular analyses would greatly enhance investigations of protein trafficking and unique protein complexes within rod photoreceptors. We present in this report two peeling methods that offer rapid, reproducible means to separate the photoreceptor cell layer of the mouse retina for quantitative protein analysis. Because the mouse retina is similar in structure/function to human peripheral retina and is rod dominant [51], our results reflect the effectiveness of the presented methods in the separation of compartments of rod photoreceptors. The purity of each compartment was verified visually (Fig. 3), as well as with Western blots using known protein markers (Figs. 2 and 4). Although we present data for separation of rod compartments, in principle both methods can be used to isolate different cellular layers of the retina with additional peels, thus broadening the impact of these new techniques on retinal research.

While both methods offered reproducible ROS isolation, it is important to note that one technique may be better suited than the other depending on the experimenter’s purpose and research questions. The filter paper peeling method produces a clean and precise ROS separation by gradual removal of the tissue surface a fraction at a time. The amount of tissue removed by each peel can be adjusted by using different types of filter paper. For example, we found that VWR grade 413 filter paper adsorbed more tissue than Whatman® Grade 1 Qualitative filter paper, requiring ~7 peels vs. ~15 peels to remove ROS. Determining the number of peels needed to isolate a specific compartment of the photoreceptor cell is critical for mastering this method. These peels can be pooled, as was done in our study, or they can be individually analyzed depending on the experimental objective.

The lyophilized retinal peeling method, on the other hand, does not allow for incremental separation within a compartment, although it, too, was effective in isolating ROS: Western results showed an enrichment of Gβ5L, a ROS marker [40], and no contamination of protein markers from other cellular compartments. The flatness of the retina and the physical pressure of the tape on the lyophilized retina are both key to the success of the isolation. Visual approximation will also play an important role in understanding which layer has been separated. For example, the peeled orange colored ROS layer often came with a uniformly thin white layer attached to it at the plane of breakage. This layer can be subsequently removed by additional tape peels, and Western blots of this layer show it to be a distinct compartment which we assigned as RIS for the following reasons: first, the appearance of cytochrome C is consistent with the presence of mitochondria in this compartment; second, the absence of Gβ5S indicates a lack of contamination from the nuclear layers underneath; finally, the +RIS compartment showed the opposite pattern of light-induced protein level changes as that of +ROS. The dramatic light-induced increase of GNAT1 level in this +RIS compartment is likely a reflection of the dissociation of GTP-loaded Gα molecules (GNAT1) from Gβγ and the disc membrane and into the (relatively) large cytoplasmic space of the RIS [26, 52, 53]. Similarly, the highest level of soluble protein ARR1 was found in the +RIS compartment in the dark-adapted retina.

Having validated the effectiveness of both peeling methods, we also investigated whether RGS9 belonged on the list of proteins that undergo light-induced protein translocation in rod cells. RGS9 and Gβ5L are part of the GTPase-accelerating protein (GAP) complex for GNAT1 which perform the critical function of transducin inactivation and recovery of the light response [39, 41, 54, 55]. According to a recent report, light caused a rapid movement of RGS9 and Gβ5L from the RIS to ROS [43]. This movement of GAP would be expected to speed the recovery of the light response and perhaps play a role in light adaptation [56]. In contrast to this report, our results using two independent peeling methods show no light-induced translocation of RGS9. This result is consistent with the known structural stability of RGS9 and Gβ5L conferred by their interaction with the membrane-anchored R9AP, which does not undergo light-triggered movement [57].

## Conclusions

Our results demonstrate the effectiveness of the two peeling protocols for separating the layered compartments of the mouse retina and their utility for investigating protein movement across these compartments.

## Abbreviations

Arr1: Arrestin
GAP: GTPase-accelerating protein
GNAT1: Transducin
GRK1: Rhodopsin kinase
IS: Inner segment
-OIS: ROS/RIS-depleted tissue
ONL: Outer nuclear layer
OPL: Outer plexiform layer
OS: Outer segment
PDE6: Phosphodiesterase 6
RIS: Rod inner segment
ROS: Rod outer segment
RPE: Retinal pigmented epithelium
RT: Room temperature
SEM: Scanning electron microscope
